# A simplified definition of diastolic function in sepsis, compared against standard definitions

**DOI:** 10.1186/s40560-019-0367-3

**Published:** 2019-02-20

**Authors:** Michael J. Lanspa, Troy D. Olsen, Emily L. Wilson, Mary Louise Leguyader, Eliotte L. Hirshberg, Jeffrey L. Anderson, Samuel M. Brown, Colin K. Grissom

**Affiliations:** 1 Critical Care Echocardiography Service, Intermountain Medical Center, 5121 S Cottonwood St, Murray, UT 84157 USA; 2 Division of Pulmonary and Critical Care Medicine, University of Utah, 30 N 1900 E, 701 Wintrobe, Salt Lake City, UT 84132 USA; 3 Department of Internal Medicine, University of Utah, 30 N 1900 E, Salt Lake City, UT 84132 USA; 4 Division of Pediatric Critical Care, Department of Pediatrics, University of Utah, 295 Chipeta Way, Salt Lake City, UT 84108 USA; 5 Intermountain Medical Center Heart Institute, 5121 S Cottonwood St, Murray, UT 84157 USA; 6 Division of Cardiology, University of Utah, 30 N 1900 E, 701 Wintrobe, Salt Lake City, UT 84132 USA

**Keywords:** Echocardiography, Septic cardiomyopathy, Diastolic dysfunction, Preload

## Abstract

**Background:**

Guidelines for grading diastolic dysfunction poorly categorize septic patients. We compared how well the American Society of Echocardiography (ASE) 2009 and 2016 definitions and a simplified definition categorized septic patients.

**Methods:**

We studied septic patients who received a transthoracic echocardiogram within 24 h of admission to an ICU. We categorized patients according to ASE 2009 and 2016 definitions and a definition using *E*/*e*’, a surrogate for left ventricular filling pressure. We assessed 28-day all-cause mortality and the presence of pre-existing diabetes, hypertension, or myocardial infarction. We tested for associations among diastolic grade, comorbidities, and outcomes using logistic regression.

**Results:**

We studied 398 patients. Mortality was 23%. The simplified definition categorized more patients than ASE 2016 (78% vs. 71%, *p* = 0.035); both definitions categorized more patients than ASE 2009 (34%, *p* < 0.001 for both comparisons). Higher grades of diastolic dysfunction were associated with hypertension (ASE 2016, simplified), myocardial infarction (ASE 2009, simplified), and diabetes (simplified). Grade of diastolic dysfunction was not associated with mortality by any definition. Of 199 patients categorized as normal by ASE 2016, 40% had an abnormal *E*/*e*′ > 9 and 7% had a severely abnormal *E*/e′ > 13.

**Conclusions:**

The ASE 2016 definition categorizes more septic patients than the ASE 2009 definition, but it does not categorize the diastolic function of a third of septic patients. ASE 2016 designates many patients with elevated *E*/*e*′ as normal. A simplified definition categorized patients with less ambiguity and is associated with relevant comorbidities.

**Electronic supplementary material:**

The online version of this article (10.1186/s40560-019-0367-3) contains supplementary material, which is available to authorized users.

## Background

Sepsis, a life-threatening organ dysfunction caused by a dysregulated host response to infection, is a common syndrome with high mortality [1]. One therapy for these patients is the administration of intravenous fluid to optimize cardiac preload [2, 3]. However, excessive fluid administration in septic patients has also been associated with increased morbidity and mortality [4–6]. Diastolic dysfunction is associated with fluid administration in the resuscitation of septic patients as well as elevated left ventricular filling pressures and is associated with mortality [7, 8]. While diastolic dysfunction is common in patients with sepsis, research is plagued by variable definitions, leading to widely disparate incidence estimates [6, 7, 9–19]. These observations highlight the need for a consistent, reproducible definition of diastolic dysfunction in sepsis.

**Fig. 1:**
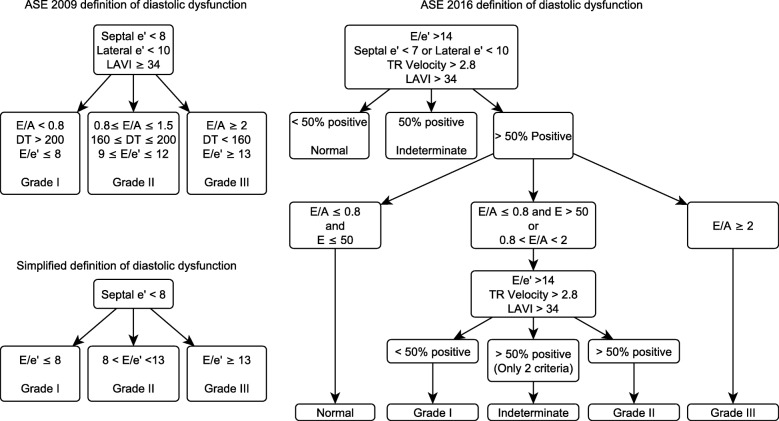
The American Society of Echocardiography (ASE) 2009 and 2016 definitions of diastolic dysfunction. *E*, velocity of early diastolic blood flow across the mitral valve. *e*′, velocity of mitral annulus at early diastole. TR, tricuspid regurgitation. LAVI, left ventricular volume index. *A*, velocity of late (atrial) diastolic blood flow across the mitral valve. DT, deceleration time of early diastolic blood flow across the mitral valve

In 2016, the American Society of Echocardiography revised its definition for diastolic dysfunction (Fig. 1) [20]. While this revision appears to be an improvement in cardiac patients, it is unknown whether it represents an improvement in the classification of diastolic function among septic patients [21]. We thus compared the American Society of Echocardiography (ASE) 2016, ASE 2009, and a previously studied simplified definition in a large cohort of septic patients. We evaluated the competing definitions on the basis of the proportion of patients categorized, proportion with ambiguous categorization, and whether categorizations are associated with relevant comorbidities or clinical outcomes.

## Methods

### Study design

This is a retrospective observational study of a prospectively identified cohort of intensive care unit (ICU) patients admitted between October 2012 and November 2015 at Intermountain Medical Center, an academic tertiary care hospital. These patients were admitted to one of two ICUs (one medical; one mixed medical, surgical, and trauma) where transthoracic echocardiogram (TTE) is routinely performed on patients with severe sepsis or septic shock at the time of ICU admission. The protocol was approved by the Intermountain Institutional Review Board (#1009957) with a waiver of informed consent.

### Patients

We screened patients between October 2012 and November 2015 admitted with severe sepsis or septic shock defined by the then-current 1992 American College of Chest Physicians/Society of Critical Care Medicine consensus criteria [22]. Patients met the criteria for inclusion if they (1) were at least 18 years of age, (2) had a clinically suspected infection, (3) had two or more systemic inflammatory response syndrome criteria, and (4) had either septic shock (systolic blood pressure < 90 mmHg despite an intravenous fluid challenge of ≥ 20 mL/kg or infusion of any dose of vasopressor medications) or severe sepsis (defined in this study as serum lactate ≥ 4 mmol/L).

### Transthoracic echocardiogram

Transthoracic echocardiograms (TTEs) were performed using a Philips iE-33 (Philips Medical Systems, Bothell, WA) machine. Patients were excluded if their TTE occurred more than 24 h after the onset of sepsis or if the image quality was poor. All TTEs were performed by a registered diagnostic cardiac sonographer. Studies were interpreted and formatted by an advanced cardiac sonographer (TDO) followed by a consensus interpretation from two level-II echocardiographers (CKG, MJL). All readers were blinded to clinical outcomes.

We measured the diastolic parameters defined in the ASE 2009 and 2016 guidelines: the ratio of early diastolic velocity of mitral inflow (*E*) to late diastolic velocity of mitral inflow (*A*), the ratio of *E* to early diastolic velocity of the average (septal and lateral) mitral annulus (*e*′), left atrial volume index (LAVI), deceleration time of early diastolic filling (DT), and tricuspid regurgitant jet velocity (TR velocity), if present. We omitted measurements pertaining to pulmonary venous inflow (Ar-A) or Valsalva maneuver (Valsalva Δ*E*/*A*) because pulmonary venous flow images are frequently of limited quality in TTE, and critically ill patients are generally unable to perform a Valsalva maneuver. Excepting TR velocity, all measurements of parameters represent the average of measurements from three consecutive cardiac cycles, when available. In rare cases when three consecutive cycles were not captured due to image quality, we used the average of two consecutive cycles. For TR velocity, we selected the highest measured velocity. In patients with sinus tachycardia or atrial fibrillation, *E* and *e*′ were determined by previously described methods [23–25]. We classified diastolic function into four grades (0, 1, 2, and 3) according to the ASE 2009, ASE 2016, and simplified definitions (Fig. 1) [26]. The simplified definition, which uses only *e*′ and *E*/*e*′, was previously developed using a machine learning approach to identify parameters that were associated with clinical outcomes in septic patients [19]. We defined a patient as categorizable if the available measurements unequivocally placed the patient in a single category. Conversely, we defined patients as uncategorizable if the available measurements were either discordant or insufficient. For the ASE 2016 definition, which allows for indeterminate status, we classified accordingly.

### Clinical data

We collected demographic information, vital signs, mechanical ventilation parameters, and vasopressor infusion rates at the time of the TTE. We converted vasopressor infusion rates of multiple vasopressors into norepinephrine-equivalent dosing, as per the previously described methods [27]. We determined the presence of comorbidities (pre-existing diabetes, hypertension, or myocardial infarction) based on discharge codes, according to Elixhauser’s method [28]. The cause of sepsis was determined by chart review (MLL). We calculated the total volume of intravenous fluid administered in 6 h before and 6 h after the TTE. We calculated the Acute Physiology and Chronic Health Evaluation II (APACHE II) [29] score at the time of ICU admission. We also measured ICU length of stay and Sequential Organ Failure Assessment (SOFA) [30] scores at the time of ICU admission and 72 h after ICU admission. Our primary clinical outcome was 28-day all-cause mortality.

### Statistical analysis

We compared the proportion of patients categorized using each definition as the primary outcome using a chi-squared test for proportions. Differences in demographic and hospitalization characteristics, echocardiographic parameters, 28-day mortality, and ICU length of stay between the diastolic grades, categorized according to each definition, were compared as secondary outcomes using the Fisher exact or Kruskal-Wallis test as appropriate. Among patients who could be categorized, we used logistic regression to assess the relationship between diastolic grade and 28-day mortality (adjusted for admission APACHE II score, mechanical ventilation, and vasopressor dose). In the same patients, we used logistic regression to assess the relationship between diastolic grade and comorbidities, including pre-existing diabetes, hypertension, and myocardial infarction. Analyses were performed using R Statistical Package, version 3.2.4 [31].

## Results

### Patient characteristics and echocardiographic measurements

We screened 1053 patients with sepsis or septic shock admitted to one of the study ICUs during the study period. Transthoracic echocardiogram was performed within the first 24 h in 398 patients who presented to these ICUs with sepsis or septic shock. In our study population of 398 patients, 67% were in shock (Table 1). The median time from ICU admission to TTE was 2.3 h. Overall 28-day mortality in the cohort was 23%. Median ICU length of stay was 1.5 days (IQR 0.8–2.8). Of the 398 study patients, 61% had measurements for all five elements of diastolic function from the ASE 2009 definition. *A*, septal *e*′, DT, LAVI, and *E* were unmeasurable in 21%, 20%, 17%, 11%, and 11% of patients, respectively. TR velocity was unmeasurable in 59% of patients. Systolic dysfunction (LVEF < 50%) was present in 13% of patients. Tachycardia (heart rate > 100 beats/min) was present in 45% of TTEs. Atrial fibrillation was present in 9% of TTEs.

**Table 1:** Demographics and clinical findings

Characteristic	*N* = 398
Age (years)	63 ± 16
Female	54%
APACHE II	26 ± 10
SOFA on admission	10 ± 4
Atrial fibrillation at time of echo	9%
On vasopressors during admission	67%
On vasopressors at time of echo	37%
Mechanically ventilated during admission	35%
Mechanically ventilated at time of echo	26%
Overall 28-day mortality	23%
Source of infection	
Pneumonia	42%
Urinary	20%
Abdominal	16%
Skin or soft tissue	12%
Endocarditis	2%
Central nervous system	< 1%
Catheter-related infection	2%
Uncertain/other	6%

Continuous data are expressed as mean ± SD, while categorical data are expressed as percentage

*APACHE II* Acute Physiology and Chronic Health Evaluation score, *SOFA* Sequential Organ Failure Assessment

### Diastolic categorization

Using the ASE 2009 definition, we categorized 107 (27%) patients as normal diastolic function and 27 (7%) as diastolic dysfunction; 264 (66%) were uncategorizable. The ASE 2016 definition categorized 199 (50%) TTEs as normal diastolic function, 83 (21%) as abnormal diastolic function, and 116 (29%) as indeterminate. The simplified definition categorized 106 (27%) patients as normal diastolic function and 203 (51%) as abnormal function; 89 (22%) were uncategorizable. Applying the ASE 2009 definition resulted in 246 patients (62%) with elements of the definition that were discordant in assigning a severity grade. By design, neither the ASE 2016 nor the simplified definition allowed for discordance: patients in the simplified definition could thus only be uncategorized on the basis of insufficient imaging data. The simplified definition categorized more patients than ASE 2016 (78% vs 71%, *p* = 0.035); both definitions categorized more patients than the ASE 2009 (34%, *p* < 0.001 for both comparisons). Relevant parameters are summarized by diastolic grade among categorizable patients for each definition in Additional file 1: Table S1.

### Conservation of diastolic grades among definitions

Although many patients were uncategorizable by the ASE 2009 definition, of the 105 (26%) patients assigned a severity grade by both ASE 2009 and ASE 2016, only five (5%) patients were categorized as having the same grade of diastolic dysfunction by both definitions (all five were grade 3). There was reasonable consistency between the ASE 2009 and ASE 2016 definitions for categorizing normal diastolic function and severe diastolic dysfunction (Table 2). While only four (4%) patients differed by two or more grades, very few patients were categorized as grade 1 or grade 2 by either definition. The simplified definition categorizations differed substantially from those of ASE 2016. Of the 228 (57%) patients assigned a severity grade by both ASE 2016 and the simplified definition, 68 (30%) patients differed by two or more grades (Table 2). The simplified definition assigned a higher diastolic severity grade than the ASE 2016 definition in 52% of patients. Fourteen patients with an *E*/*e*′ > 13 were categorized as grade 1 or 0 by the ASE 2016 definition. Of 199 patients categorized as normal by ASE 2016, 40% had an abnormal *E*/*e*′ > 9 and 7% had a severely abnormal *E*/*e*′ > 13. Also of interest is that 52% of the patients who were uncategorizable by the simplified definition (meaning the patient had insufficient imaging data to calculate an *E*/*e*′) were classified as normal by the ASE 2016 definition. In contrast, 45% of patients who were indeterminate by the ASE 2016 definition were classified as grade 3 (*E*/*e*′ > 13) by the simplified definition.

**Table 2:** Comparison of categorization by definition

ASE grade	ASE 2016 Grade 0	ASE 2016 Grade 1	ASE 2016 Grade 2	ASE 2016 Grade 3
ASE 2009 grade 0	87	2	1	1
ASE 2009 grade 1	7	0	0	0
ASE 2009 grade 2	2	0	0	0
ASE 2009 grade 3	0	0	0	5
Simplified grade 0	88	2	1	1
Simplified grade 1	2	3	0	0
Simplified grade 2	39	11	1	1
Simplified grade 3	24	3	40	12

### Association of diastolic grade with clinical outcomes and pre-existing comorbidities

We observed no significant differences in mortality (irrespective of adjustment for admission APACHE II scores, mechanical ventilation, and vasopressor dose) or in organ-failure-free days between any of the diastolic severity grades produced using any of the three definitions (Table 3 and Additional file 2: Table S2). While we noted trends in ICU length of stay, the problem of the competing risk of death makes inference difficult. Using the simplified definition, patients categorized with normal diastolic function received more intravenous fluid during the subsequent 6 h than those with diastolic dysfunction (median 498 vs 0 mL, *p* = 0.05). In the ASE 2009 and 2016 definitions, higher grades of diastolic dysfunction were associated with hypertension (OR 1.66, 95% CI 1.09–2.75, *p* = 0.03, and OR 1.30, 95% CI 1.00–1.72, *p* = 0.06, respectively) and history of myocardial infarction (OR 2.07, 95% CI 1.38–3.16, *p* < 0.001, and 1.81, 95% CI 1.37–2.41, *p* < 0.001), but not diabetes. In the simplified definition, higher grades of diastolic dysfunction were associated with hypertension (OR 1.34, 95% CI 1.13–1.61, *p* = 0.001), myocardial infarction (OR 1.75, 95% CI 1.37–2.31, *p* < 0.001), and diabetes (OR 1.25, 95% CI 1.05–1.51, *p* = 0.02). These associations are detailed in Additional file 3: Table S3. We also noted that patients categorized as grade 1 diastolic dysfunction using the simplified definition had greater APACHE II scores and had received less intravenous fluid in 6 h preceding the TTE compared with other diastolic grades (Additional file 1: Table S1).

**Table 3:** Multivariable logistic regression for 28-day mortality, with covariates of diastolic grade (adjusted for admission APACHE II score, mechanical ventilation at time of echocardiogram, and vasopressor dose at time of echocardiogram) for all three definitions. For patients on multiple vasopressors, all doses were converted to norepinephrine-equivalent doses, as per the previously described methods [27]

Covariate	Odds ratio	95% confidence interval	*p* value
ASE 2009			
Diastolic grade (0–3)	1.00	(0.56, 1.64)	0.99
APACHE II	1.14	(1.08, 1.23)	< 0.001
Mechanical ventilation	0.60	(0.19, 1.70)	0.35
Vasopressor dose (per 1 mg/kg/min increase norepinephrine equivalent)	1.02	(1.00, 1.04)	0.08
ASE 2016			
Diastolic grade (0–3)	1.37	(0.97, 1.94)	0.07
APACHE II	1.13	(1.09, 1.18)	< 0.001
Mechanical ventilation	0.43	(0.18, 0.98)	0.05
Vasopressor dose (per 1 mg/kg/min increase norepinephrine equivalent)	1.01	(1.00, 1.03)	0.12
Simplified			
Diastolic grade (0–3)	1.16	(0.91, 1.49)	0.24
APACHE II	1.12	(1.08, 1.17)	< 0.001
Mechanical ventilation	0.37	(0.16, 0.81)	0.02
Vasopressor dose (per 1 mg/kg/min increase norepinephrine equivalent)	1.02	(1.01, 1.04)	0.02

Among the categorizable patients, mechanical ventilation status during the TTE was not associated with grade for the ASE 2009 or ASE 2016 definitions (*p* = 0.80 and *p* = 0.39, respectively, Additional file 1: Table S1). However, lower simplified diastolic grades were associated with greater receipt of mechanical ventilation (*p* = 0.01). Similarly, whether a patient was receiving vasopressors during the TTE was not associated with diastolic grade for the ASE 2009 or ASE 2016 definitions (*p* = 0.08 and *p* = 0.33, respectively), but lower simplified diastolic grades were associated with greater receipt of vasopressors (Additional file 1: Table S1, *p* = 0.02). Patients receiving vasopressors had a lower *E*/*e*′ (10.4, IQR 8.2–13.9) than those who were not (11.7, IQR 8.6–16.4, *p* = 0.01).

## Discussion

It appears that a simplified definition of diastolic dysfunction categorizes more patients than the ASE 2009 and 2016 definitions and has reasonable correlation with relevant comorbidities. The ASE 2016 definition categorized more patients with sepsis and septic shock than the ASE 2009 definition. Our findings support and expand upon a smaller cohort of septic patients [32]. Several elements of the ASE 2009 definitions were not measurable in critically ill septic patients. The omission of these elements in both the simplified and the ASE 2016 definition seems reasonable, as some elements, including left atrial volume index and deceleration time, do not appear to be as clinically important in septic patients, where patients can have acute changes in ventricular compliance related to rapid changes in preload and adrenergic tone [19, 33]. We note that these limitations may also apply outside sepsis: in a cohort of 200 patients with clinical heart failure, ASE 2016 was unable to categorize half of the patients [34].

While the simplified definition was associated with relevant comorbidities and had some association with intravenous fluid volumes received, it is unknown whether the simplified definition is clinically superior to the ASE 2016 definition, and categorization alone may be an insufficient indicator of superiority. There is no “gold standard” by which one can easily compare these definitions, because invasive measurements of diastolic function are infeasible or uncommon in septic patients. Despite this, *E*/*e*′ correlates well with left ventricular end-diastolic pressure in septic patients [35–37]. *E*/*e*′ may also aid in decisions on volume loading, diuresis, and liberation from mechanical ventilation [35–39]. We observed no differences in mortality among any of the definitions, although the simplified definition was associated with mechanical ventilation and vasopressor receipt and dose. It is possible that the grade of diastolic dysfunction may not directly influence early clinical outcomes among septic patients. We noted that patients with grade 1 diastolic dysfunction had the highest APACHE II scores, suggesting that diastolic grade may not be linearly associated with disease severity.

One finding of interest is that 63 of the 153 patients (41%) classified as normal by ASE 2016 had an elevated *E*/*e*′, which would be grade 2 or 3 using the simplified definition (Table 2). It seems incorrect to designate these patients as normal. The characteristics of the ASE 2016 definition demonstrate that almost half of those categorized as indeterminate had an *E*/*e*′ > 13. Conversely, half of the patients who were uncategorizable by the simplified definition were “normal” according to ASE 2016, as the ASE can allow for categorization without assessing *E*/*e*′ in some cases.

Since the simplified definition relies on solely two measurements, the mitral inflow velocity and the medial mitral annulus velocity, it appears to have some use in the critically ill patient where image quality may limit other measurements needed for the ASE 2009 or 2016 definition. Due to its simplicity, it can be easily internalized for rapid bedside assessment by the clinician with a moderate amount of ultrasound training. The exclusion of tricuspid velocity may avoid misclassification of patients with pulmonary disease. The simplified definition may identify patients with acute fluid overload that might have been classified as normal due to a normal left atrial volume index or a borderline *e*′. Despite these potential advantages, there still is need for further evaluation and validation.

There are challenges in attempting to simplify a diastolic function, which is a complex assessment, into a single number. Nevertheless, the simplified definition may offer some value with regard to its categorization of normal and grade 1 diastolic dysfunction. We observed that patients with grade 1 dysfunction received less intravenous fluid prior to their echocardiogram, confirming our prior observations in a different cohort of septic patients [7]. We also observed that patients who had normal diastolic function using the simplified and ASE 2016 definitions received more fluid following their echocardiograms than those with abnormal diastolic function. While the treating physician was not necessarily blinded to the echocardiogram, we believe that most of the immediate fluid management decisions were made without knowing or acting on the diastolic function, as many of the treating physicians did not have access to diastolic grading for the subsequent 6-h window. The association of diastolic function with fluid receipt suggests that the simplified definition may have value as an assessment to consider in future research involving fluid management in a critically ill patient with sepsis.

This study is the largest to our knowledge of diastolic function in sepsis. This study differs from prior studies that typically performed the TTE much later after the onset of sepsis or were limited to patients receiving mechanical ventilation [15, 17, 18, 32, 40]. In contrast, we performed echocardiography early in the course of sepsis (median time 2.3 h from ICU admission), and we included patients regardless of the receipt of mechanical ventilation. Therefore, this study may be less affected by survivorship bias than prior studies that performed echocardiography among survivors 24 h after onset of sepsis. The inclusion of spontaneously breathing patients increases this study’s generalizability, as substantial numbers of patients with septic shock are managed without mechanical ventilation.

### Limitations

Our definitions for severe sepsis and septic shock [22], although appropriate at the time of study, were subsequently replaced by the Sepsis-3 definitions [41], meaning that this cohort of patients may not precisely align with patient cohorts defined with sepsis or septic shock in more recent or future publications. The majority of our patients were medical ICU patients, and our findings may not be generalizable to ICUs with different patient populations. We did not use all of the parameters included in the ASE 2009 definition (Ar-A, Valsalva ∆*E*/*A*) due to the challenges of acquiring these measurements in a critically ill population [26]. The simplified definition uses only the septal *e*′, rather than an average of both septal and lateral *e*′, and might therefore be more susceptible to right ventricular dysfunction. Our study captured a single echocardiographic study during the early course of sepsis, rather than several studies; echocardiographic parameters may be affected by heart rate, preload, and arrhythmia, which may vary during an ICU stay. There is a possibility of selection bias, as not all septic patients admitted to the ICU received an echocardiogram; it is possible that this study population may represent a sicker population than the general septic population.

## Conclusions

The ASE 2016 definition does not categorize diastolic function in almost a third of septic patients and also designates some patients with apparently elevated filling pressures as normal. A simplified definition based on *E*/*e*′ categorizes more septic patients and correlates with key comorbidities commonly associated with diastolic dysfunction. We believe that this simplified definition will appeal to the practicing intensivist, but further prospective testing is warranted to validate and extend these observations.

## Additional files

Additional file 1: **Table S1.** Incidence and clinical characteristics of diastolic dysfunction by specific definitions employed. Continuous data are displayed as medians and interquartile ranges, while categorical data are displayed as *n* (%). APACHE II, Acute Physiology and Chronic Health Evaluation score. BMI, body mass index. IVF, intravenous fluid. SOFA, Sequential Organ Failure Assessment (DOCX 32 kb)

Additional file 2: **Table S2.** Multivariable linear regression for organ-failure-free days (composite of renal, hepatic, coagulation, and cardiovascular components of the Sequential Organ Failure Assessment score, out of 14 days) (DOCX 18 kb)

Additional file 3: **Table S3.** Univariate regression models for comorbidities using diastolic grade of 0–3 (DOCX 16 kb)

## Abbreviations

*A*: Velocity of late (atrial) diastolic blood flow across the mitral valve
APACHE II: Acute Physiology and Chronic Health Evaluation score, version 2
ASE: American Society of Echocardiography
BMI: Body mass index
DT: Deceleration time of early diastolic blood flow across the mitral valve
*E*: Velocity of early diastolic blood flow across the mitral valve
*e*′: Velocity of mitral annulus at early diastole
ICU: Intensive care unit
IVF: Intravenous fluid
LAVI: Left ventricular volume index
SOFA: Sequential organ failure assessment
TR: Tricuspid regurgitation
TTE: Transthoracic echocardiogram
