# Supplementary material for: A simplified definition of diastolic function in sepsis, compared against standard definitions
Source: J Intensive Care. 2019 Feb 20;7:14. doi: 10.1186/s40560-019-0367-3 (PMC6381727; doi:10.1186/s40560-019-0367-3)
Supplement: Supplementary file 1 — Table S1. Incidence and clinical characteristics of diastolic dysfunction by specific definitions employed. Continuous data are displayed as medians and interquartile ranges, while categorical data are displayed as n (%). APACHE II, Acute Physiology and Chronic Health Evaluation score. BMI, body mass index. IVF, intravenous fluid. SOFA, Sequential Organ Failure Assessment (DOCX 32 kb) [file 40560_2019_367_MOESM1_ESM.docx]

**Table S1** Incidence and clinical characteristics of diastolic dysfunction by specific definitions employed. Continuous data are displayed as medians and interquartile ranges, while categorical data are displayed as n (%). APACHE II, Acute Physiology and Chronic Health Evaluation score. BMI, Body Mass Index. IVF, Intravenous Fluid. SOFA, sequential organ failure assessment.

| Characteristic | Grade 0 | Grade I | Grade II | Grade III | p value |
| --- | --- | --- | --- | --- | --- |
| ASE 2009 definition (of 134 categorizable patients) | | | | | |
| Patients | 107 (80%) | 11 (8%) | 4 (3%) | 12 (9%) | - |
| Age (years) | 55 (38, 65) | 69 (65, 77) | 70 (61, 74) | 74 (61, 81) | **<0.001** |
| Female | 55 (51%) | 6 (55%) | 3 (75%) | 5 (42%) | 0.76 |
| Hypertension | 49 (46%) | 7 (64%) | 2 (50%) | 9 (75%) | 0.07 |
| Diabetes | 35 (33%) | 3 (27%) | 0 (0%) | 6 (50%) | 0.27 |
| Myocardial infarction | 12 (11%) | 3 (27%) | 2 (50%) | 6 (50%) | **0.001** |
| BMI (kg/m^2^) | 28 (24, 36) | 35 (23, 42) | 30 (22, 82) | 29 (26, 30) | 0.84 |
| APACHE II | 25 (17, 34) | 31 (22, 33) | 32 (21, 33) | 29 (20, 32) | 0.74 |
| SOFA on admission | 11 (7, 13) | 9 (8, 12) | 12 (10, 13) | 8 (7, 12) | 0.61 |
| Atrial fibrillation at time of echo | 11 (10%) | 1 (9%) | 1 (25%) | 3 (25%) | 0.22 |
| On vasopressors during admission | 79 (74%) | 5 (46%) | 3 (75%) | 8 (67%) | 0.24 |
| On vasopressors at time of echo | 50 (47%) | 2 (18%) | 0 (0%) | 6 (50%) | 0.08 |
| Mechanically ventilated during admission | 47 (44%) | 4 (36%) | 2 (50%) | 3 (25%) | 0.63 |
| Mechanically ventilated at time of echo | 29 (27%) | 4 (36%) | 1 (25%) | 2 (17%) | 0.80 |
| IVF given in 6h prior to TTE (mL) | 980 (0, 2275) | 350 (0, 2000) | 880 (569, 1250) | 1425 (0, 2308) | 0.98 |
| IVF given in 6h after TTE (mL) | 495 (0, 1896) | 0 (0, 255) | 110 (0, 415) | 951 (12, 1194) | 0.36 |
| E (cm/s) | 93 (79, 106) | 54 (36, 74) | 68 (61, 71) | 109 (94, 130) | **0.001** |
| A (cm/s) | 80 (61, 100) | 74 (57, 93) | 71 (55, 79) | 28 (15, 42) | **0.002** |
| Average e’ (cm/s) | 11.1 (10.1, 12.6) | 6.1 (5.7, 6.4) | 7.5 (6.6, 7.6) | 6.5 (5.8, 6.7) | **<0.001** |
| E/e’ (average) | 8.4 (6.7, 10.1) | No observations | 10.4 (9.7, 11.0) | 21.1 (15.8, 21.8) | **<0.001** |
| DT (ms) | 188 (158, 222) | 278 (252, 297) | 178 (166, 191) | 130 (111, 139) | **<0.001** |
| E/A | 1.2 (0.9, 1.4) | 0.6 (0.6, 0.7) | 1.0 (0.9, 1.1) | 4.1 (3.4, 9.6) | **<0.001** |
| LAVI (ml/m^2^) | 27 (21, 33) | 40 (38, 55) | 95 (71, 119) | 41 (38, 51) | **<0.001** |
| Ejection fraction | 63 (57, 69) | 68 (57, 70) | 54 (48, 55) | 47 (32, 52) | **<0.001** |
| 28-day Mortality | 23 (22%) | 2 (18%) | 3 (75%) | 2 (17%) | 0.12 |
| ICU length of stay (days) | 1.7 (0.8, 3.4) | 2.1 (0.6, 2.7) | 0.9 (0.2, 1.5) | 1.3 (0.7, 4.4) | 0.40 |
| ASE 2016 definition (of 282 categorizable patients) | | | | | |
| Patients | 199 (70%) | 23 (8%) | 44 (16%) | 16 (6%) | - |
| Age (years) | 61 (49, 71) | 65 (45, 73) | 71 (62, 75) | 72 (58, 82) | **0.001** |
| Female | 104 (52%) | 7 (30%) | 29 (66%) | 8 (50%) | 0.05 |
| Hypertension | 112 (56%) | 10 (44%) | 31 (71%) | 11 (69%) | 0.05 |
| Diabetes | 65 (33%) | 6 (26%) | 17 (39%) | 9 (56%) | 0.13 |
| Myocardial infarction | 24 (12%) | 7 (30 %) | 18 (41%) | 5 (31%) | **<0.001** |
| BMI (kg/m^2^) | 27 (24, 35) | 25 (23, 27) | 28 (24, 32) | 29 (27, 33) | 0.09 |
| APACHE II | 25 (18, 34) | 26 (17, 38) | 22 (17, 28) | 23 (14, 31) | 0.33 |
| SOFA on admission | 10 (7, 13) | 9 (7, 12) | 7 (5, 11) | 8 (6, 9) | **0.01** |
| Atrial fibrillation at time of echo | 17 (9%) | 0 (0%) | 1 (2%) | 2 (13%) | 0.18 |
| On vasopressors during admission | 141 (71%) | 16 (70%) | 27 (61%) | 8 (50%) | 0.25 |
| On vasopressors at time of echo | 86 (43%) | 9 (39%) | 14 (32%) | 4 (25%) | 0.33 |
| Mechanically ventilated during admission | 79 (40%) | 10 (44%) | 9 (21%) | 3 (19%) | **0.03** |
| Mechanically ventilated at time of echo | 55 (28%) | 8 (35%) | 8 (18%) | 3 (19%) | 0.39 |
| IVF given in 6h prior to TTE (mL) | 1000 (0, 2000) | 0 (0, 1050) | 271 (0, 1625) | 250 (0, 2662) | 0.16 |
| IVF given in 6h after TTE (mL) | 222 (0, 1549) | 0 (0, 900) | 0 (0, 1000) | 675 (0, 1000) | 0.11 |
| E (cm/s) | 83 (71, 102) | 57 (47, 79) | 113 (96, 134) | 109 (93, 145) | **<0.001** |
| A (cm/s) | 84 (65, 101) | 74 (54, 94) | 101 (82, 122) | 36 (29, 43) | **<0.001** |
| Average e’ (cm/s) | 9.4 (7.4, 11.2) | 6.9 (5.6, 7.9) | 6.1 (5.0, 7.1) | 6.1 (5.3, 6.6) | **<0.001** |
| E/e’ (average) | 9.2 (7.8, 11.2) | 9.5 (7.9, 10.0) | 18.0 (16.6, 21.7) | 19.6 (16.1, 24.5) | **<0.001** |
| DT (ms) | 197 (165, 230) | 180 (149, 215) | 188 (149, 220) | 170 (130, 198) | 0.07 |
| E/A | 1.0 (0.8, 1.3) | 0.8 (0.6, 1.1) | 1.1 (0.9, 1.5) | 2.8 (2.3, 3.8) | **<0.001** |
| LAVI (ml/m^2^) | 24 (19, 30) | 21 (19, 28) | 40 (31, 46) | 39 (32, 45) | **<0.001** |
| Ejection fraction | 64 (59, 70) | 42 (36, 47) | 63 (52, 70) | 47 (40, 58) | **<0.001** |
| 28-day Mortality | 41 (21%) | 4 (17%) | 9 (21%) | 6 (38%) | 0.45 |
| ICU length of stay (days) | 1.6 (0.8, 3.0) | 1.7 (0.8, 2.8) | 1.0 (0.8, 1.9) | 0.8 (0.4, 1.4) | **0.02** |
| Simplified definition (of 309 categorizable patients) | | | | | |
| Patients | 106 (34%) | 8 (3%) | 64 (21%) | 131 (42%) | - |
| Age (years) | 56 (41, 66) | 63 (50, 72) | 68 (57, 74) | 72 (62, 80) | **<0.001** |
| Female | 57 (54%) | 3 (38%) | 22 (34%) | 92 (70%) | **<0.001** |
| Hypertension | 51 (48%) | 6 (75%) | 38 (59%) | 89 (68%) | **0.005** |
| Diabetes | 32 (30%) | 3 (38%) | 17 (27%) | 60 (46%) | **0.01** |
| Myocardial infarction | 8 (8%) | 1 (13%) | 15 (23%) | 41 (31%) | **<0.001** |
| BMI (kg/m^2^) | 27.4 (24.0, 34.2) | 24.7 (21.7, 29.6) | 27.6 (24.5, 32.7) | 28.5 (23.4, 33.1) | 0.55 |
| APACHE II | 25 (16, 34) | 37 (33, 42) | 23 (18, 31) | 23 (18, 30) | **0.03** |
| SOFA on admission | 10 (7, 13) | 11.5 (9, 13) | 9 (7, 12) | 8 (6, 11) | **0.01** |
| Atrial fibrillation at time of echo | 10 (9%) | 0 (0%) | 5 (8%) | 11 (8%) | 0.97 |
| On vasopressors during admission | 81 (76%) | 6 (75%) | 42 (66%) | 77 (59%) | **0.03** |
| On vasopressors at time of echo | 52 (49%) | 4 (50%) | 22 (34%) | 39 (30%) | **0.02** |
| Mechanically ventilated during admission | 46 (43%) | 6 (75%) | 19 (30%) | 33 (25%) | **0.002** |
| Mechanically ventilated at time of echo | 30 (28%) | 6 (75%) | 13 (20%) | 29 (22%) | **0.01** |
| IVF given in 6h prior to TTE (mL) | 1000 (0, 2962) | 0 (0, 168) | 1050 (0, 2662) | 180 (0, 1975) | **0.002** |
| IVF given in 6h after TTE (mL) | 498 (0, 1700) | 0 (0, 1138) | 143 (0, 1000) | 0 (0, 1000) | 0.19 |
| E (cm/s) | 93 (79, 108) | 48 (38, 53) | 72 (57, 80) | 102 (85, 125) | **<0.001** |
| A (cm/s) | 81 (64, 102) | 62 (55, 68) | 83 (65, 97) | 94 (71, 113) | **0.003** |
| Average e’ (cm/s) | 10.5 (9.4, 12.0) | 7.1 (5.6, 7.5) | 7.2 (6.3, 7.8) | 6.2 (5.2, 7.3) | **<0.001** |
| E/e’ (average) | 8.5 (6.9, 10.2) | 7.0 (6.5, 7.8) | 9.6 (8.6, 11.0) | 16.5 (13.7, 19.6) | **<0.001** |
| DT (ms) | 192 (160, 223) | 254 (193, 299) | 189 (156, 218) | 193 (154, 235) | 0.48 |
| E/A | 1.2 (0.8, 1.4) | 0.8 (0.7, 0.9) | 0.9 (0.7, 1.0) | 1.1 (0.8, 1.7) | **<0.001** |
| LAVI (ml/m^2^) | 27 (22, 34) | 17 (14, 25) | 22 (18, 30) | 31 (24, 42) | **<0.001** |
| Ejection fraction | 63 (58, 69) | 47 (39, 62) | 60 (52, 66) | 63 (53, 71) | **0.04** |
| 28-day Mortality | 25 (24%) | 1 (13%) | 15 (23%) | 29 (22%) | 0.96 |
| ICU length of stay (days) | 1.7 (0.9, 3.9) | 1.7 (0.5, 3.1) | 1.6 (0.8, 2.7) | 1.3 (0.7, 2.7) | 0.18 |
