# Supplementary material for: A simplified definition of diastolic function in sepsis, compared against standard definitions
Source: J Intensive Care. 2019 Feb 20;7:14. doi: 10.1186/s40560-019-0367-3 (PMC6381727; doi:10.1186/s40560-019-0367-3)
Supplement: Supplementary file 2 — Table S2. Multivariable linear regression for organ-failure-free days (composite of renal, hepatic, coagulation, and cardiovascular components of the Sequential Organ Failure Assessment score, out of 14 days) (DOCX 18 kb) [file 40560_2019_367_MOESM2_ESM.docx]

**Table S2** Multivariable Linear Regression for Organ Failure Free days (composite of renal, hepatic, coagulation, and cardiovascular components of the Sequential Organ Failure Assessment score, out of 14 days. Covariates of diastolic grade (adjusted for admission APACHE II score, mechanical ventilation at time of echocardiogram, and vasopressor dose at time of echocardiogram) for all three definitions. For patients on multiple vasopressors, all doses were converted to norepinephrine-equivalent doses, per previously described methods.^33^ P values are unadjusted for multiple hypothesis testing.

| Covariate | Coefficient | 95% Confidence Interval | p-value |
| --- | --- | --- | --- |
| ASE 2009 |  |  |  |
| Diastolic Grade (0-3) | 0.28 | -0.40, 0.97 | 0.41 |
| APACHE II | -0.23 | -0.30, -0.16 | <0.001 |
| Mechanical ventilation | 2.06 | 0.49, 3.63 | 0.01 |
| Vasopressor dose  (per 1 mcg/kg/min increase norepinephrine equivalent) | -0.44 | -0.07, -0.02 | 0.003 |
| ASE 2016 |  |  |  |
| Diastolic Grade (0-3) | -0.04 | -0.46, 0.38 | 0.85 |
| APACHE II | -0.23 | -0.28, -0.18 | <0.001 |
| Mechanical ventilation | 1.68 | 0.56, 2.8 | 0.003 |
| Vasopressor dose  (per 1 mcg/kg/min increase norepinephrine equivalent) | -0.03 | -0.05, -0.02 | <0.001 |
| Simplified |  |  |  |
| Diastolic Grade (0-3) | 0.06 | -0.25, 0.37 | 0.71 |
| APACHE II | -0.21 | -0.26, -0.16 | <0.001 |
| Mechanical ventilation | 1.10 | -0.03, 2.25 | 0.06 |
| Vasopressor dose  (per 1 mcg/kg/min increase norepinephrine equivalent) | -0.03 | -0.05, -0.01 | <0.001 |
