# Supplementary material for: A simplified definition of diastolic function in sepsis, compared against standard definitions
Source: J Intensive Care. 2019 Feb 20;7:14. doi: 10.1186/s40560-019-0367-3 (PMC6381727; doi:10.1186/s40560-019-0367-3)
Supplement: Supplementary file 3 — Table S3. Univariate regression models for comorbidities using diastolic grade of 0–3 (DOCX 16 kb) [file 40560_2019_367_MOESM3_ESM.docx]

**Table S3** Univariate regression models for comorbidities using diastolic grade of 0 – 3.

| Comorbidity | Odds Ratio | 95% confidence interval | P value |
| --- | --- | --- | --- |
| **ASE 2009** | | | |
| Hypertension | 1.66 | 1.09-2.75 | 0.03 |
| Myocardial Infarction | 2.07 | 1.38-3.16 | <0.001 |
| Diabetes | 1.17 | 0.79-1.73 | 0.42 |
|  |  |  |  |
| **ASE 2016** | | | |
| Hypertension | 1.30 | 1.00-1.72 | 0.06 |
| Myocardial Infarction | 1.81 | 1.37-2.41 | <0.001 |
| Diabetes | 1.21 | 0.95-1.57 | 0.13 |
|  |  |  |  |
| **Simplified** | | | |
| Hypertension | 1.34 | 1.13-1.61 | 0.001 |
| Myocardial Infarction | 1.75 | 1.37-2.31 | <0.001 |
| Diabetes | 1.25 | 1.05-1.51 | 0.02 |
